# Assessment of Renal Function in Patients with Chronic Kidney Disease with and without Hypothyroidism

**DOI:** 10.4314/ejhs.v35i1.4

**Published:** 2025-01

**Authors:** Pujitha Mallina, Vinay Rajan, Eswar Kumar, Gullipalli Prasad

**Affiliations:** 1 M.Pharm-Pharmacy Practice, A.U. College of Pharmaceutical Sciences, Andhra University, Visakhapatnam, 530003; 2 A.U. College of Pharmaceutical Sciences, Andhra University, Visakhapatnam, 530003; 3 Department of Nephrology, Andhra Medical College, Visakhapatnam, 530002

**Keywords:** Chronic Kidney Disease, CKD, Creatinine, eGFR, Kidney Function, Renal Markers, Thyroid Dysfunction, Hypothyroidism

## Abstract

**Background:**

Hypothyroidism is a common endocrine disorder with a bi-directional relationship to Chronic Kidney Disease (CKD), presenting a notable complication in CKD patients. This study aimed to explore the impact of hypothyroidism on kidney function in CKD patients.

**Materials and Methods:**

This study included 150 participants, with 110 CKD patients without hypothyroidism and 40 CKD patients with hypothyroidism. The participants were further categorized into stages 3, 4, and 5 based on their estimated Glomerular Filtration Rate (eGFR). They were followed for three consecutive months at intervals of 28 ± 3 days, 57 ± 3 days, and 86 ± 3 days. Clinical and demographic data, including age, gender, serum creatinine, serum urea, Blood Urea Nitrogen (BUN), eGFR, and serum sodium, potassium, and chloride levels, were assessed over time. Data analysis was performed using GraphPad Prism, with a significance level set at 0.05%.

**Results:**

In CKD patients with hypothyroidism, serum creatinine (P = 0.0002), serum urea (P = 0.0046), and BUN (P = 0.0042) levels were significantly higher, while eGFR (P < 0.0001) was lower compared to CKD patients without hypothyroidism. Potassium levels were significantly elevated in CKD patients with hypothyroidism (P = 0.0001), whereas no significant difference was observed in serum sodium (P = 0.0802) or chloride (P = 0.2089) levels.

**Conclusion:**

This study concludes that CKD patients with hypothyroidism experience a more significant decline in kidney function compared to CKD patients without hypothyroidism.

## Introduction

Chronic Kidney Disease (CKD) affects approximately 13.4% of the global population and is currently the 10^th^ leading cause of death, projected to become the 5^th^ by 2040. In 2017, approximately 843.6 million people worldwide were affected by CKD (1, 2).

Thyroid hormones significantly influence kidney function, with both pre-renal effects on the cardiovascular system and Renal Blood Flow (RBF), and direct renal effects on eGFR, tubular processes, and hormonal regulation of renal function (3, 4). Thyroid abnormalities, particularly hypothyroidism, are prevalent in both non-dialysis-dependent and dialysis-dependent CKD patients, contributing to higher mortality rates compared to the general population (5). Hypothyroidism, a prevalent endocrine disorder, is recognized as a notable complication in CKD patients with a bidirectional relationship (6, 7, 8). The burden of hypothyroidism is high in CKD patients, although many cases may remain latent and undiagnosed (9, 10, 11, 12, 13).

Hypothyroidism in CKD is often a result of altered thyroid hormone metabolism, where the conversion of thyroxine (T4) to triiodothyronine (T3) is reduced due to decreased activity of the type 1 deiodinase enzyme. Additionally, thyroid hormone transport can be disrupted by changes in Thyroxine-Binding Globulin (TBG), leading to a relative deficiency of active thyroid hormones (14, 15, 16). This can affect CKD patients by reducing RBF, decreasing renal responsiveness to beta-adrenergic stimuli, inhibiting the Renin-Angiotensin-Aldosterone System (RAAS), and impairing filtration.

## Materials and Methods

**Sampling design and size**: This prospective, observational study involved 150 participants divided into two groups:

- **Arm-A**: 110 CKD-only patients
- **Arm-B**: 40 CKD patients with hypothyroidism

Participants were categorized into stages 3, 4, and 5 of CKD based on eGFR. They were further divided according to the duration of their CKD history: less than 6 months, 6–12 months, and more than 12 months. Arm-A included patients from all stages, while Arm-B only included stage 5 CKD patients.

**Study site and population**: This study was conducted in the Nephrology Department of a tertiary care hospital. Patients with urinary tract obstruction, renal calculi, lupus nephritis, chronic pyelonephritis, chronic use of analgesics, aminoglycosides, immunosuppressants, or a history of hypothyroidism before CKD were excluded.

**Data collection**: Data were collected at three time points: baseline, 28 ± 3 days, 57 ± 3 days, and 86 ± 3 days. Demographic and clinical data, including age, gender, CKD duration, CKD stage, serum creatinine, urea, BUN, eGFR, sodium, potassium, and chloride, were assessed.

**Statistical analysis**: Data were analyzed using GraphPad Prism, with a significance level of 0.05%. Descriptive statistics were presented as mean ± Standard Deviation (SD) with 95% Confidence Intervals (CI). Categorical variables were analyzed using the chi-square test, and ANOVA was used to compare data within arms. The t-test was applied for group comparisons at each visit.

**Ethical considerations**: The study followed ethical guidelines as outlined in the Declaration of Helsinki. Approval was obtained from the Institutional Ethics Committee (IEC) of King George Hospital, Visakhapatnam. Informed consent was obtained from all participants.

## Results

**Figure 1:**
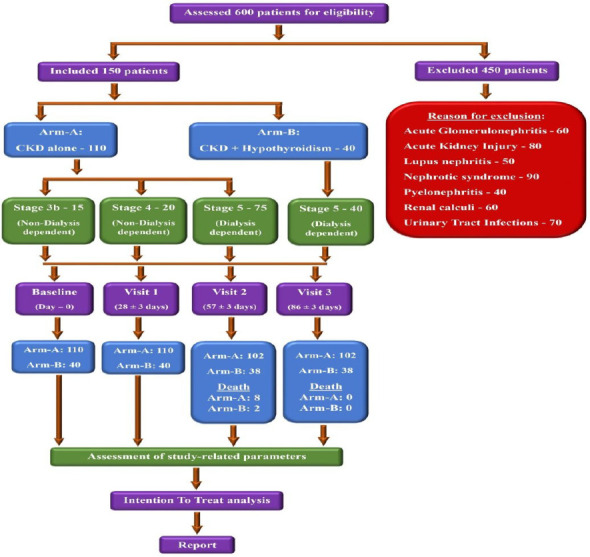
Overview of the study

The study included 150 participants, with 110 in Arm-A (CKD-only) and 40 in Arm-B (CKD with hypothyroidism) (Figure 1).

**Demographics**: In Arm-A, 72.73% of participants were male and 27.27% female. In Arm-B, 62.50% were male and 37.50% female. The mean age for Arm-A was 51.7 ± 13.2 years and for Arm-B, 49.5 ± 11.7 years. The majority in Arm-A were aged 41-50 years (31 subjects), while in Arm-B, the majority were aged 51–60 years (14 subjects).

**CKD stages**: In Arm-A, 15 subjects were in stage 3b, 20 in stage 4, and 75 in stage 5. Arm-B included only stage 5 CKD patients.

**Renal markers:** At baseline, serum creatinine, urea, and BUN levels were significantly higher in Arm-B, while eGFR levels were significantly lower compared to Arm-A. Potassium levels were significantly higher in Arm-B (P = 0.0001). However, no significant differences were observed in sodium (P = 0.0802) and chloride (P = 0.2089) levels (Table 1).

**Table 1:** Stage-wise Renal Function Tests between Arm-A and Arm-B

Renal Function Tests	Arm-A Mean ± SD (CI)	Arm-B Mean ± SD (CI)	P-value^a^	Stage 5		P-value^a^

				Arm-A Mean ± SD (CI)	Arm-B Mean ± SD (CI)	
Serum creatinine	6.03 ± 3.5	7.1 ± 1.8	**0.0002**	7.7 ± 3.0	7.1 ± 1.8	**0.0295**
(mg/dL)	(5.69 - 6.36)	(6.85 - 7.44)		(7.37 - 8.07)	(6.85 - 7.44)	
Serum urea	89.9 ± 43.6	100.9 ± 33.3		105.3 ± 44.1	100.9 ± 33.3	
(mg/dL)	(85.81 - 94.13)	(95.65 - 106.2)	**0.0046**	(100.2 - 110.4)	(95.65 - 106.2)	0.2809
Serum BUN	41.9 ± 20.4	47.1 ± 15.5		49.1 ± 20.6	47.1 ± 15.5	
(mg/dL)	(39.98 - 43.89)	(44.66 - 49.59)	**0.0042**	(46.76 - 51.57)	(44.66 - 49.59)	0.2833
eGFR	15.1 ± 11.1	8.4 ± 2.4		8.5 ± 3.3	8.4 ± 2.4	
(mL/min/1.73m^2^)	(14.09 - 16.22)	(8.02 - 8.80)	**< 0.0001**	(8.11 - 8.89)	(8.02 - 8.80)	0.7842
Serum sodium	138.7 ± 3.2	138.2 ± 3.3		138.5 ± 3.6	138.2 ± 3.3	
(mmol/L)	(138.4 - 139.0)	(137.6 - 138.7)	0.0802	(138.1 - 139.0)	(137.6 - 138.7)	0.3317
Serum potassium	4.5 ± 0.7	4.7 ± 0.6	**0.0001**	4.6 ± 0.7	4.7 ± 0.6	0.3442
(mmol/L)	(4.43 - 4.57)	(4.65 - 4.86)		(4.61 - 4.77)	(4.65 - 4.86)	
Serum chloride	101.4 ± 4.1	102.0 ± 4.7	0.2089	101.4 ± 4.2	102.0 ± 4.7	0.1848
(mmol/L)	(101.1 - 101.8)	(101.2 - 102.7)		(100.9 - 101.9)	(101.2 - 102.7)	

*Arm-A: Patients with only CKD; Arm-B: Patients with CKD and Hypothyroidism: a: unpaired t-test; BUN: Blood Urea Nitrogen; eGFR: estimated Glomerular Filtration Rate; CI: Confidence Interval*.

**Figure 2:**
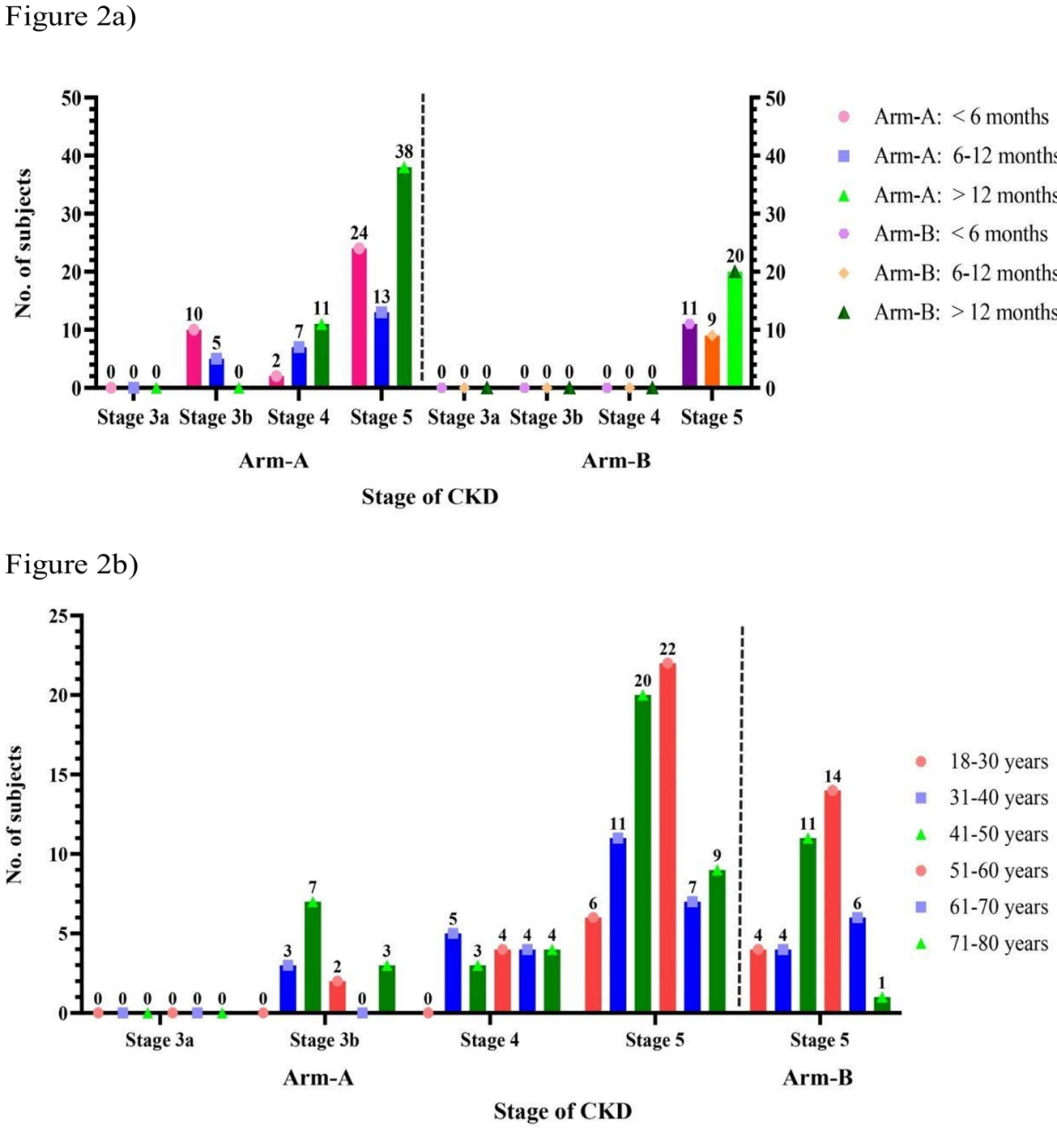
**a**-Stage-wise and duration of history of CKD in Arm-A & Arm-B; **b**- Stage-wise and Age-wise distribution in Arm-A & Arm-B

**Effect of duration of CKD history**: Significant differences were observed in serum creatinine, potassium, and chloride levels in both arms, particularly in subjects with a history of CKD longer than 12 months (Table 2). Figure 2a illustrates the stage wise distribution of study participants with respect to their duration of CKD history.

**Table 2:** Renal Function Tests based on the duration of history of CKD in Arm-A & Arm-B

Renal Function Tests	Duration of CKD	Stage 5		P-value^a^

		Arm-A	Arm-B	
Serum creatinine	< 6 months	8.5 ± 3.3	7.8 ± 2.3	0.1856
(mg/dL)	6-12 months	7.2 ± 2.0	7.4 ± 2.2	0.6792
	> 12 months	7.3 ± 2.9	6.6 ± 1.2	**0.0493**
Serum Urea	< 6 months	118.9 ± 45.29	113.8 ± 39.74	0.5285
(mg/dL)	6-12 months	106.1 ± 47.9	110.1 ± 28.6	0.6759
	> 12 months	96.4 ± 39.9	90.6 ± 28.2	0.2517
Serum BUN	< 6 months	55.5 ± 21.1	53.1 ± 18.5	0.5274
(mg/dL)	6-12 months	49.5 ± 22.6	51.0 ± 13.3	0.7257
	> 12 months	45.0 ± 18.6	42.3 ± 13.1	0.2510
eGFR	< 6 months	7.4 ± 3.1	7.6 ± 2.6	0.6294
(mL/min/1.73m^2^)	6-12 months	8.7 ± 2.6	7.8 ± 1.9	0.0913
	> 12 months	9.1 ± 3.5	9.0 ± 2.4	0.8810
Serum sodium	< 6 months	137.2 ± 3.4	137.5 ± 2.6	0.7100
(mmol/L)	6-12 months	138.6 ± 2.7	137.9 ± 4.9	0.4034
	> 12 months	139.3 ± 3.8	138.7 ± 2.8	0.1983
Serum potassium	< 6 months	4.5 ± 0.7	4.3 ± 0.5	0.3999
(mmol/L)	6-12 months	4.5 ± 0.5	4.8 ± 0.5	**0.0324**
	> 12 months	4.8 ± 0.7	4.9 ± 0.6	0.5229
Serum chloride	< 6 months	101.0 ± 4.9	100.2 ± 4.7	0.3795
(mmol/L)	6-12 months	101.2 ± 4.5	101.4 ± 4.5	0.8322
	
	> 12 months	101.6 ± 3.6	103.1 ± 4.5	**0.0096**

*Arm-A: Patients with only CKD*

*Arm-B: Patients with CKD and Hypothyroidism*

*a: unpaired t-test*

*BUN: Blood Urea Nitrogen*

*eGFR: estimated Glomerular Filtration Rate*

**Changes over time**: Significant differences in serum creatinine, BUN, eGFR, and potassium levels were observed between Arm-A and Arm-B at various visits, particularly at visits 2 and 3. However, no significant differences were found in serum sodium and chloride levels (Table 3).

**Table 3:** The Renal Function Tests at each visit for Arm-A & Arm-B

Renal Function Tests	Arm	Baseline	visit 1	visit 2	visit 3	P-Value^b^

		Mean ± SD				
Serum creatinine	Arm-A	6.2 ± 3.5	6.2 ± 3.4	5.9 ± 3.4	5.6 ± 3.4	**0.0483**
(mg/dL)	Arm-B	6.4 ± 1.5	6.9 ± 1.4	7.3 ± 1.7	7.9 ± 2.3	**0.0004**
	P-value^a^	0.7297	0.2173	**0.0196**	**0.0004**	-
Serum urea	Arm-A	98.5 ± 48.0	91.2 ± 42.8	87.0 ± 40.8	82.2 ± 40.8	**0.002**
(mg/dL)	Arm-B	95.9 ± 27.4	101.2 ± 33.9	101.6± 34.4	105.3 ± 37.6	0.2029
	P-value^a^	0.7457	0.1897	0.0539	**0.0029**	-
Serum BUN	Arm-A	46.0 ± 22.4	42.6 ± 20.1	40.2 ± 19.4	38.4 ± 19.0	**0.0001**
(mg/dL)	Arm-B	44.8 ± 12.8	47.2 ± 15.8	47.4 ± 16.0	49.1 ± 17.6	0.2089
	P-value^a^	0.7435	0.195	**0.0451**	**0.0030**	-
eGFR	Arm-A	15.2 ± 12.3	14.6 ± 11.0	15.1 ± 10.7	15.6 ± 10.4	0.1839
(mL/min/1.73m^2^)	Arm-B	9.3 ± 2.4	8.6 ± 2.3	8.0 ± 2.3	7.6 ± 2.4	**0.0002**
	P-value^a^	**0.0028**	**0.0007**	**0.0001**	**< 0.0001**	-
Serum sodium	Arm-A	139.2 ± 4.1	138.4 ± 3.2	138.5 ± 2.8	138.9 ± 2.7	0.0636
(mmol/L)	Arm-B	137.3 ± 4.0	138.5 ± 2.6	138.3 ± 2.8	138.7 ± 3.7	0.1347
	P-value^a^	**0.0156**	0.8799	0.7234	0.7498	-
Serum potassium	Arm-A	4.5 ± 0.8	4.4 ± 0.6	4.5 ± 0.6	4.4 ± 0.6	0.6497
(mmol/L)	Arm-B	4.7 ± 0.8	4.7 ± 0.5	4.7 ± 0.6	4.7 ± 0.5	0.8641
	P-value^a^	0.1674	**0.0151**	0.1021	**0.0110**	-
Serum chloride	Arm-A	102.2 ± 4.3	100.9 ± 4.3	101.1 ± 3.7	101.4 ± 3.8	**0.0006**
(mmol/L)	Arm-B	101.7 ± 5.1	101.9 ± 4.4	101.8 ± 4.3	102.5 ± 5.0	0.6305
	
	P-value^a^	0.4849	0.2193	0.3737	0.1609	-

*Arm-A: Patients with only CKD*

*Arm-B: Patients with CKD and Hypothyroidism*

*a: unpaired t-test*

*BUN: Blood Urea Nitrogen*

*eGFR: estimated Glomerular Filtration Rate*

*b: ANOVA*

**Age group analysis**: Statistically significant differences were observed in serum creatinine, urea, BUN, and eGFR levels across different age groups in both arms, with the most significant differences noted in the 41–50 and 51–60 age groups. Figure 2b illustrates the stage wise distribution of study participants with respect to their age.

**Thyroid function**: Mean serum TSH levels were significantly higher in Arm-B compared to Arm-A (P < 0.0001). Serum T4 and T3 levels also differed, with significant changes in T4 (P = 0.0110) but no significant differences in T3 (P = 0.0560) (Table 4).

**Table 4:** Thyroid Function Tests in Arm-A and Arm-B

Arm	TSH	T4	T3
	(µIU/mL)	(ug/dL)	(ng/dL)
	
		Mean ± SD (CI)	
**Arm-A**	2.4 ± 1.5	9.4 ± 2.2	0.9 ± 0.2
	(2.1 – 2.7)	(8.9 – 9.8)	(0.8 – 1.0)
**Arm-B**	25.9 ± 51.8	8.1 ± 3.3	6.3 ± 13.6
	(9.3 – 42.5)	(6.8 – 9.5)	(- 8.0 – 20.6)

**P-value^a^**	**< 0.0001**	**0.0110**	0.0560

*T4 – thyroxine*

*T3 - triiodothyronine*

*Arm-A: Patients with only CKD*

*Arm-B: Patients with CKD and Hypothyroidism*

*CI: Confidence Interval*

*a: Mann-Whitney test*

**Gender and renal function**: Males showed more compromised kidney function than females across both arms.

## Discussion

According to our study results, renal function is more compromised in patients with both CKD and hypothyroidism, as evidenced by elevated serum creatinine, serum urea, and BUN levels, compared to CKD patients without hypothyroidism. These findings align with Jaiswal et al. (2018), who reported significantly higher serum creatinine levels in patients with thyroid dysfunction compared to control subjects (17). However, these results contrast with those of Intisar Al Fahdi et al. (2022), who found no significant difference between CKD patients with and without hypothyroidism (18).

Thyroid dysfunction is more prevalent in patients with an eGFR < 15 mL/min/1.73m^2^ (Lakesh Singla et al., 2023), and has been shown to contribute to further kidney function deterioration (19). The decline in eGFR is attributed to various factors, including increased sensitivity to adrenergic stimulation, reduced renin release, diminished renin-angiotensin system activity, and decreased renal parenchymal growth, which limits glomerular surface area for filtration. Furthermore, hypothyroidism exacerbates eGFR decline by reducing cardiac output, increasing peripheral vascular resistance, causing intrarenal vasoconstriction, and impeding the renal response to vasodilators such as vascular endothelial growth factor and insulin-like growth factor-1.

It is well established that kidney function worsens as CKD progresses, with elevated serum creatinine levels being a key indicator. Interestingly, in our study, mean serum creatinine levels were slightly lower in CKD patients compared to CKD patients with hypothyroidism. This marginal difference may be explained by the more extensive dialysis sessions undergone by hypothyroid CKD patients. Additionally, patients in Arm-B exhibited a significant decline in eGFR from baseline to visit 3, further supporting findings by Christiaan L. et al. (2019), who noted that hypothyroidism is associated with a more pronounced decrease in eGFR compared to patients with normal thyroid function (20).

In contrast to renal function markers like serum creatinine, urea, and BUN, the levels of electrolytes such as sodium, potassium, and chloride were within normal ranges. This suggests that, while hypothyroidism significantly impacts kidney function, its effect on electrolyte levels may be more limited. Hypothyroidism contributes to renal dysfunction through mechanisms such as decreased renal plasma flow, impaired glomerular filtration, and reduced creatinine excretion, which ultimately leads to elevated serum creatinine levels. Consequently, thyroid dysfunction correlates with the severity of CKD.

In stage 5 CKD, a comparative analysis based on CKD duration revealed notable differences in various biochemical parameters. Specifically, a significant difference in mean serum creatinine levels was observed only in subjects with a CKD history longer than 12 months, indicating that prolonged CKD may influence creatinine levels. Conversely, no significant differences were found in serum urea, BUN, eGFR, serum sodium, or chloride levels across different CKD history durations. However, a significant difference in mean serum potassium levels was observed in subjects with a CKD history of 6-12 months, suggesting a specific impact during this intermediate stage of CKD. This highlights the importance of considering CKD duration when interpreting biochemical markers, as variations may be observed at different stages.

Further research is needed to explore the underlying mechanisms behind these findings and their implications for patient management. Our study underscores that renal function deteriorates more significantly in CKD patients with hypothyroidism compared to those with CKD alone. The limitations of this study include a small sample size, unequal distribution of participants across groups, and a relatively short follow-up period.

In conclusion, our study found that CKD patients with hypothyroidism had higher serum creatinine, urea, and BUN levels compared to CKD patients without hypothyroidism. These findings suggest that hypothyroidism negatively affects renal physiology by reducing renal blood flow (RBF), impairing glomerular filtration, and elevating serum creatinine, urea, and BUN levels. Furthermore, eGFR levels were lower in patients with both CKD and hypothyroidism, indicating a correlation between hypothyroidism and CKD severity. Although there was no significant difference in serum sodium or chloride levels, potassium levels were significantly higher in patients with both conditions, further emphasizing the impact of hypothyroidism. This study concludes that renal function deteriorates more in CKD patients with hypothyroidism compared to those with CKD alone.
